# Effect of Freshly Isolated Bone Marrow Mononuclear Cells and Cultured Bone Marrow Stromal Cells in Graft Cell Repopulation and Tendon-Bone Healing after Allograft Anterior Cruciate Ligament Reconstruction

**DOI:** 10.3390/ijms22062791

**Published:** 2021-03-10

**Authors:** Cheng-Chang Lu, Cheng-Jung Ho, Hsuan-Ti Huang, Sung-Yen Lin, Shih-Hsiang Chou, Pei-Hsi Chou, Mei-Ling Ho, Yin-Chun Tien

**Affiliations:** 1 Department of Orthopedics, Kaohsiung Municipal Siaogang Hospital, Kaohsiung Medical University, Kaohsiung 812, Taiwan; cclu0880330@gmail.com; 2 Department of Orthopedics, College of Medicine, Kaohsiung Medical University, Kaohsiung 807, Taiwan; hthuang@kmu.edu.tw (H.-T.H.); tony8501031@gmail.com (S.-Y.L.); arthroscopy_pc@yahoo.com.tw (P.-H.C.); 3 Graduate Institute of Medicine, College of Medicine, Kaohsiung Medical University, Kaohsiung 807, Taiwan; 4 Regenerative Medicine and Cell Therapy Research Center, Kaohsiung Medical University, Kaohsiung 807, Taiwan; homelin@kmu.edu.tw; 5 Department of Orthopedics, Kaohsiung Medical University Hospital, Kaohsiung Medical University, Kaohsiung 807, Taiwan; rick_free@mail2000.com.tw (C.-J.H.); stanelychou@gmail.com (S.-H.C.); 6 Department of Orthopedics, Kaohsiung Municipal Ta-Tung Hospital, Kaohsiung Medical University, Kaohsiung 807, Taiwan; 7 Department of Physiology, College of Medicine, Kaohsiung Medical University, Kaohsiung 807, Taiwan

**Keywords:** ACL reconstruction, allograft, bone marrow mononuclear cells, bone marrow stromal cells, ligamentization, tendon to bone healing

## Abstract

Graft cell repopulation and tendon-bone tunnel healing are important after allograft anterior cruciate ligament reconstruction (ACLR). Freshly isolated bone marrow mononuclear cells (BMMNCs) have the advantage of short isolation time during surgery and may enhance tissue regeneration. Thus, we hypothesized that the effect of intra-articular BMMNCs in post-allograft ACLR treatment is comparable to that of cultured bone marrow stromal cells (BMSCs). A rabbit model of hamstring allograft ACLR was used in this study. Animals were randomly assigned to the BMMNC, BMSC, and control groups. Fresh BMMNCs isolated from the iliac crest during surgery and cultured BMSCs at passage four were used in this study. A total of 1 × 10^7^ BMMNCs or BMSCs in 100 µL phosphate-buffered saline were injected into the knee joint immediately after ACLR. The control group was not injected with cells. At two and six weeks post operation, we assessed graft cell repopulation with histological and cell tracking staining (PKH26), and tendon-bone healing with histological micro-computed tomography and immunohistochemical analyses for collagen I and monocyte chemoattractant protein-1 (MCP1). At two weeks post operation, there was no significant difference in the total cell population within the allograft among the three groups. However, the control group showed significantly higher cell population within the allograft than that of BM cell groups at six weeks. Histological examination of proximal tibia revealed that the intra-articular delivered cells infiltrated into the tendon-bone interface. Compared to the control group, the BM cell groups showed broader gaps with interfacial fibrocartilage healing, similar collagen I level, and higher MCP1 expression in the early stage. Micro-CT did not reveal any significant difference among the three groups. BMMNCs and BMSCs had comparable effects on cell repopulation and interfacial allograft-bone healing. Intra-articular BM cells delivery had limited benefits on graft cell repopulation and caused higher inflammation than that in the control group in the early stage, with fibrocartilage formation in the tendon-bone interface after allograft ACLR.

## 1. Introduction

Knee injuries, particularly in the anterior cruciate ligament (ACL), are the most common sports-related injuries. Currently, ACL reconstruction (ACLR) with implanted graft is the standard treatment for ACL rupture. The major concerns after ACLR graft implantation are graft maturation and tendon-bone tunnel interfacial healing [1,2]. Cell repopulation is critical in all phases of tendon graft remodeling, and insufficient cell repopulation within the ACL graft may induce degeneration or micro-ruptures of the graft during the postoperative period [3]. Fast interfacial healing of grafted tendon and bone tunnel is needed to prevent graft pull-out and allow early joint rehabilitation. Therefore, accelerating cell repopulation in implanted grafts and tendon-bone tunnel interfacial healing are important for preventing graft failure, increasing graft strength, and improving clinical outcome of ACLR.

The types of grafts used in ACLR include autograft, allograft, and artificial graft. Autograft provides the benefit of faster incorporation without the risks of immune rejection and disease transmission. Nevertheless, autograft harvesting may lead to pain in anterior knee, patellar fracture, weakness of quadriceps, knee flexion weakness, and saphenous nerve injury [4,5]. The use of artificial graft in ACLR has a clinical outcome similar to that of autograft in short and mid-term follow up; however, the development of joint synovitis, high failure rate, and poor graft incorporation are some of the major concerns associated with its use [6,7]. Hence, the application of allograft in ACLR has become increasingly popular, as it provides the advantages of eliminating donor site morbidity, availability of multiple grafts, and shorter operative time [8]. However, the use of decellularized allograft tendons results in delayed healing due to insufficient population of living cells, slow migration of native cells, and inferior biomechanical strength compared to autografts [9,10]. Therefore, to enhance graft regeneration and tendon-bone interfacial healing, there is need to accelerate decellularized allograft revitalization with increased cellularity.

Cell therapy has been applied to enhance tissue regeneration, and bone marrow stromal cells (BMSCs) are the most commonly used cell source for allograft tendon regeneration both in vitro and in vivo [11,12]. Ning et al. [11] implanted rat BMSCs into decellularized tendons and showed that the implanted cells proliferated with high tenogenic gene expression. Lu et al. [12] seeded BMSCs through the lateral slit to revitalize the decellularized tendon and found that the BMSCs were redistributed in the whole decellularized tendon, which had higher collagen, tenogenic, and MMP gene expression than normal tendon. Nevertheless, there are limitations to the clinical application of BMSCs, such as the time and techniques needed for cell expansion prior to use, high cost, pathogen contamination, and risk of generic alterations [13]. Bone marrow mononuclear cells (BMMNCs), another attractive cell source for tissue regeneration, have the advantage of being easy to isolate from fresh bone marrow (BM) in a short time period (within one hour), without cell expansion. BMMNCs have been used in the treatment of myocardium infarction, limb ischemia, ischemia-reperfusion muscle injury, stroke, as well as tissue-engineered vascular grafts and bone defect reconstruction [14,15,16]. BMMNCs also possess the ability to adhere to biomaterials, promote angiogenesis, and enhance intrinsic healing through the production of cytokines [17,18,19]. To date, there are no reports in the literature that directly compare between the enhancing effects of intra-articular injection with BMMNCs and BMSCs after allograft ACLR.

For clinical application, the effect of cell therapy on enhancing cell repopulation and tendon-bone interfacial healing after allograft ACLR requires further understanding. We hypothesized that freshly isolated BMMNCs have similar or better effects on enhancing cellular repopulation and tendon-bone tunnel interfacial healing after allograft ACLR, compared to cultured BMSCs. In this study, we evaluated the effects of freshly isolated BMMNCs and cultured BMSCs on enhancement of allograft cell repopulation and interfacial healing in vivo in a rabbit model of ACLR using hamstring allograft.

## 2. Results

### 2.1. Analysis of Cell Repopulation in the Intra-Articular Graft

#### 2.1.1. Cell Repopulation within Allograft

**Figure 1:**
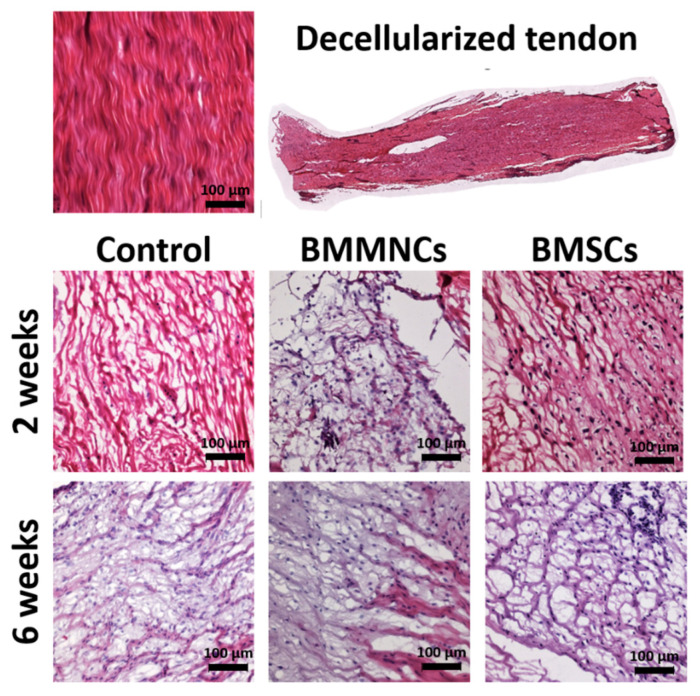
Representative pictures of decellularized tendon graft in the control, BMMNC, and BMSC groups at 2 weeks and 6 weeks after operation. BMMNCs: Bone Marrow Mononuclear Cells; BMSCs: Bone Marrow Stromal Cells. Bar = 100 µm.

Before implantation, the decellularized tendon graft contained well-oriented fibers but no cells. Two weeks after ACLR, the grafts in the control, BMMNC, and BMSC groups had cells and disoriented collagen fibers. Six weeks after allograft implantation, all three groups showed abundant cells with improved collagen fiber alignment (Figure 1).

#### 2.1.2. Higher Total Cell Population within Allograft in the Control Group Than in the Bone Marrow Cells-Seeded Groups

**Figure 2:**
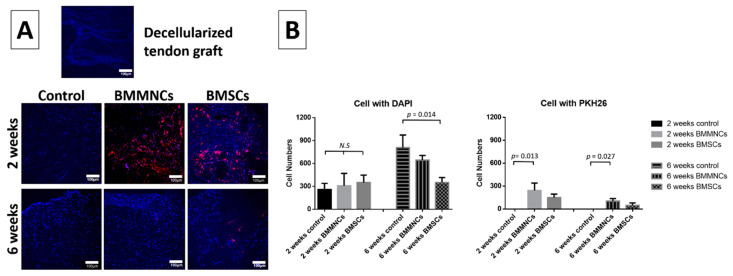
(**A**) The histological pictures of DAPI/PKH26 staining in decellularized tendon graft, control, BMMNC, and BMSC groups. (**B**) The total cell number (DAPI; blue) and seeded cells (PKH26; red) in the control, BMMNC, and BMSC groups 2 and 6 weeks after operation. *n* = 3 per group. Blue: DAPI (cell nucleus); Red: PKH26 (seeded cells). White bar = 100 µm. N.S. indicate no statistically significant difference between groups.

To monitor the injected cells in the allograft sites, we used the membrane-based cell tracker PKH26. The pre-implanted decellularized tendon graft showed limited cell nuclei (blue DAPI stain) and no injected cells (red PKH26 stain). The total cell population within allograft tissues increased in all three groups from two to six weeks (*n* = 3 per group). Although, the total cell number in the control population was lower than the BM cell-injected groups at two weeks post surgery, there was no significant difference. At six weeks post surgery, the control group had the highest total cell among the three groups. The number of injected cells (PKH26) decreased from two to six weeks (Figure 2). At two weeks, the fluorescent signals in the cells were detectable. After six weeks, cells with membrane-based trackers in the plasma membrane were diluted due to cell proliferation and the fluorescent signals were weaker than the threshold of detectable signal.

### 2.2. Analysis of Tendon-Bone Tunnel Interfacial Healing in the Proximal Tibia

#### 2.2.1. The Presence of Intra-Articular-Injected Bone Marrow Cells in the Interface between the Tendon-Bone Tunnel

**Figure 3:**
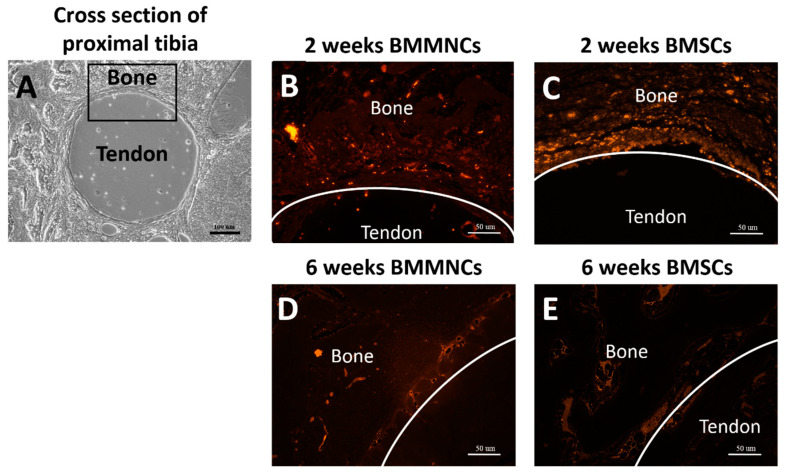
(**A**) Representative picture of the cross section of proximal tibia showed the implanted allograft tendon and surrounding bone. The black box demonstrated the observed area of tendon-bone interface. (**B**,**C**) Intra-articular-injected BMMNCs and BMSCs (PKH26 stained in red) were infiltrated in the tendon-bone interface two weeks after operation. (**D**,**E**) Few BMMNCs and BMSCs were found in the tendon-bone interface six weeks after operation. Black bar: 100 µm; White bar: 50 µm.

The intra-articular-injected BM cells (stained with PKH26 in red; BMMNCs (Figure 3B) and BMSCs (Figure 3C)) infiltrated into the interface between the tendon-bone tunnel after two weeks post operation. At six weeks post operation, few injected BM cells were found in the tendon-bone interface (Figure 3D,E). 

#### 2.2.2. Interfacial Fibrocartilage Formation in the Tendon-Bone Tunnel in Bone Marrow Cells-Injected Groups

**Figure 4:**
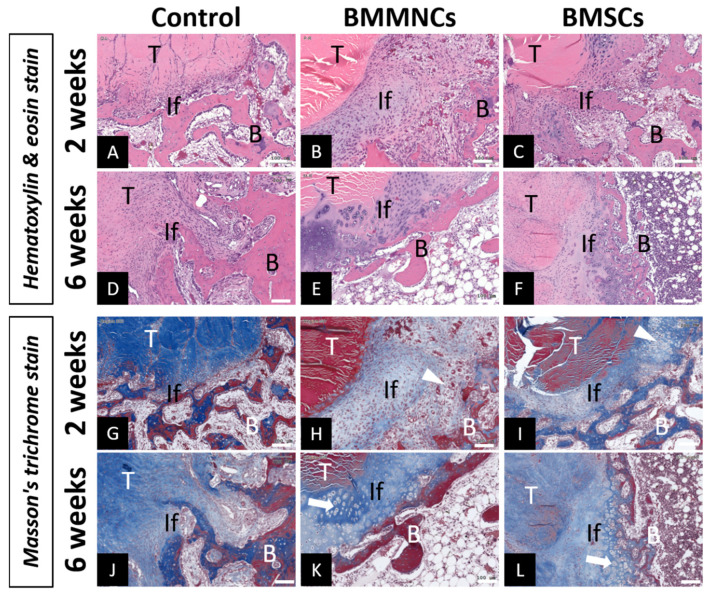
Haematoxylin and eosin (H&E) and Masson trichrome staining of tendon-bone interface in the control, BMMNC, and BMSC groups. The control group showed smaller interface gaps at both two (**A**,**G**) and six weeks (**D**,**J**), and more maturated and reorganized collagen fibers penetrating the tendon at six weeks without obvious junction (**D**,**J**). The BM-injected groups presented broader interface with fibrocartilage healing at two weeks (**B**,**C**,**H**,**I**) and six weeks (**E**,**F**,**K**,**L**). White arrow head at two weeks (**H**,**I**) and white arrow at six weeks (**K**,**L**) indicate the fibrocartilage formation area. T: Allograft tendon; B: Bone; If: Interface; Bar = 100 µm.

Compared to the relatively narrow interface (not quantified) in the control group, more cells infiltrated and formed localized areas of chondroid-like cells (Figure 4H (BMMNCs) and Figure 4I (BMSCs); white arrowhead) between the tendon-bone tunnel in the BM cell groups after two weeks post surgery. The dense tendon cortex was also visible at two weeks post surgery. At six weeks post surgery, the tendon cortex was unidentifiable in the control and BM cell injection (BMMNC and BMSC) groups, with different interfacial patterns. In the control group, collagen fiber infiltration into the grafted tendon was observed in the interface, indicating progressive maturation and reorganization of fibrous tissue. In contrast, broader fibrocartilage zone (Figure 4K (BMMNCs) and Figure 4L (BMSCs); white arrow) with collagen fiber infiltration was observed in the tendon-bone interface of the BMMNC and BMSC groups, indicating transitional healing. These findings were shown in Figure 4.

#### 2.2.3. Micro-CT Revealed No Significant Differences between the Control and Bone Marrow Cells-Seeded Groups

**Figure 5:**
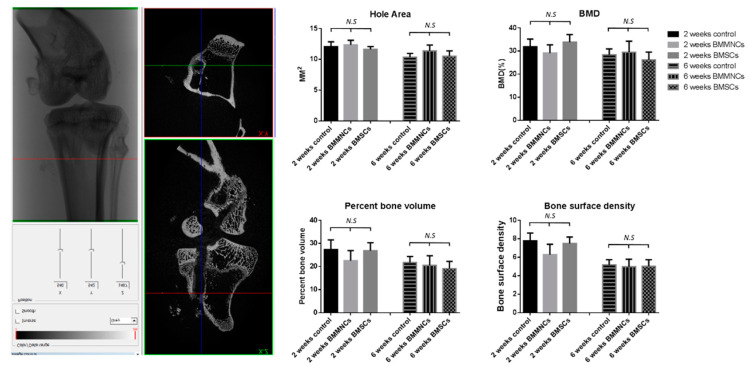
Micro-CT analysis of proximal tibia specimens. The figures show the result of bone tunnel hole area, BMD, percent bone volume, and bone surface density among the control, BMMNC, and BMSC groups at two and six weeks after operation. *n* = 4 in control group, *n* = 5 in BM cells-injected groups at both two and six weeks. N.S. indicate no statistically significant difference between groups.

Using micro-CT, we observed that the hole area decreased from two weeks to six weeks without significant difference between the control and BM cells-seeded groups. At both two and six weeks, no significant differences in BMD, percent bone volume, or bone surface density were observed among the three groups (Figure 5; *n* = 4 in control group, *n* = 5 in BM cells-injected groups at both two and six weeks). 

#### 2.2.4. Upregulated MCP1 Expression in the Bone Marrow Cells-Seeded Groups

**Figure 6:**
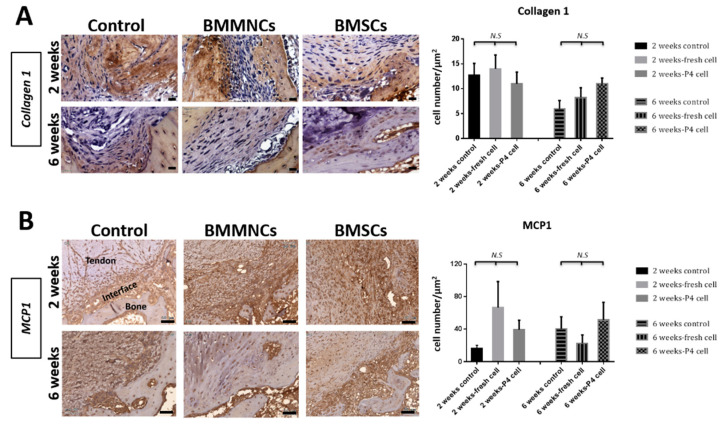
Representative immunohistochemistry images revealed collagen I (**A**) and MCP1 (**B**) expression in control, BMMNC, and BMSC groups at two and six weeks. Bar = 50 µm. *n* = 3 in each group at both two and six weeks. N.S. indicate no statistically significant difference between groups.

IHC revealed that the expression level of collagen I declined at six weeks compared to that at two weeks in all the three groups. There was no significant difference in collagen I expression level among the three groups at both two and six weeks (Figure 6A; *n* = 3 in each group at both two and six weeks). To investigate the inflammation status after injection with BM cells, we monitored the expression of MCP1, an important cytokine that modulates the infiltration and migration of macrophages/monocytes to the site of inflammation [20,21]. At two weeks, the BM cells-injected groups exhibited higher MCP1 expression levels than the control group without significant difference. At six weeks, the expression of MCP1 was variable among the three groups, but without significant difference (Figure 6B; *n* = 3 in each group at both two and six weeks).

## 3. Discussion

Cell therapy has been proposed to improve graft maturation and tendon-bone tunnel healing after ACLR. However, the effects of bone marrow cells obtained from different sources in allograft ACLR are not well investigated. This study evaluated the enhancing effects of freshly isolated BMMNCs and cultured BMSCs on graft recellularization and tendon-bone interfacial healing in a rabbit allograft ACLR model. At two and six weeks post operation, BMMNC and BMSC showed similar capabilities of cell repopulation within the intra-articular allograft. However, in this study, we did not observe any benefit of intra-articular BM cell injection; indeed, the control group had a higher total cell population in the graft than the BM cells-injected groups at six weeks post operation. BMMNCs and BMSCs resulted in similar tendon-bone interfacial healing patterns with broader gap and progressive fibrocartilage formation, in contrast to fiber reorganization in the control group. IHC revealed similar collagen I levels and higher inflammation in the BM cells-injected groups than in the control group at two weeks post operation. Micro-CT showed no differences in the hole area and bone density among the three groups. These findings indicated that intra-articular injection of BM cells induced more inflammation in the early stages, with limited benefits in allograft cell repopulation, and promoted fibrocartilage transition zone development between the allograft tendon and bone tunnel.

The clinical outcomes of allograft in ACLR is controversial. For example, a systemic review comparing hamstring autograft and soft-tissue allograft in ACLR revealed no significant differences in the outcomes [22]. Another long-term follow-up (>10 years) study reported significantly higher ACLR failure rate for allograft (26.5%) compared to that for autograft (8.3%) [23]. Previous studies on the efficacy of cell therapy in animal models of ACLR have shown variable results owing to different factors, including cell sources, implanted graft (autograft, allograft, xenograft), applied cell numbers, animal species, studied area (graft or tendon bone interface), delivery time (immediate, days, or weeks after ACLR), and delivery method (intra-articular injection, intra-tunnel injection, or cell sheet) [24,25,26]. Our study focused on the use of intra-articular cell injection for cell repopulation in grafts and tendon-to-bone tunnel healing after allograft ACLR. In contrast to previous studies, our study focused on the use of freshly isolated bone marrow-derived cells, which are easy to prepare for one-time surgeries. In addition, we performed intra-articular injection immediately after operation to simulate clinical situations. Interestingly, intra-articular injection of both freshly isolated BMMNCs and cultured BMSCs exhibited similar effects in our study, with limited effect on graft cell repopulation and different interfacial fibrocartilage healing patterns compared to that of the control group, which was not injected with cells.

Clinical images and previous studies have shown that both recellularization and revascularization are delayed and biomechanical strength is inferior in allografts compared to autografts after ACLR [9,10,27]. In the rabbit allograft ACLR model used in our study, we injected BM-derived cells to allow self-proliferation and differentiation, and promote the secretion of paracrine factors that attract and enhance the activity and differentiation of surrounding cells. The cells present within the allograft not only limited the proliferation of the seeded cells but also the migration of surrounding cells. We found abundant cell repopulation in the allograft at six weeks compared to two weeks post operation in all three groups. After six weeks, plentiful cells were found in the intra-articular allograft tissue in the control group, indicating that surrounding cells migrated into the allograft tissue without the injection of exogenous cells. Interestingly, the total cell population was highest in the control group compared to BM cell groups at six weeks post operation. In this study, intra-articular BM cell injection did not enhance allograft cell repopulation, indicating that it did not significantly promote and indeed, might have even impaired peripheral cell migration into the allograft.

After ACLR, the grafted tendon was initially integrated with the fibrous tissue in the bone tunnel wall. The interfacial fibrous tissue progressively matured into Sharpey’s fibers in indirect healing or developed into layered chondral formations at the tendon-bone interface in direct healing [28]. BMSCs with different delivery methods enhanced the interfacial healing in the tendon-bone tunnel after ACLR through transitional fibrocartilage zone formation [24,29]. Soon et al. [29] applied fibrin sealant to Achilles tendon allograft coated with cultured autogenous P1 BMSCs and reported improved interfacial healing in the bone tunnel with a mature fibrocartilage zone. Kanazawa et al. [24] applied autologous BMSCs, with the carrier transplanted between the autograft tendon and tibia bone pit, in an ACLR rabbit model and observed that treatment with BMSC resulted in progressive maturation of active chondroid cells layered in the interface from four to eight weeks. Nevertheless, the exact mechanism of interfacial fibrocartilage formation after BM cell therapy is unclear. In the present study, we first demonstrated that cells could migrate and infiltrate into the interface between the grafted tendon and bone tunnel after intra-articular injection. Histological analysis revealed that the injection of BM cells resulted in the formation of a broader fibrocartilage transitional zone anchored to tendons, resembling direct healing; this was obviously different from the healing pattern in the control group, which exhibited more organized fiber infiltration into the graft. In this study, higher MCP1 expression was observed at two weeks post operation, indicating early inflammation after additional BM cell delivery, which might explain the early gap and later fibrocartilage formation in the tendon-bone tunnel after allograft ACLR. This study demonstrated that intra-articular injection of both freshly isolated BMMNCs and cultured BMSCs had similar capabilities for forming interfacial fibrocartilage after ACLR [24,29].

BM-derived cells showed a biphasic role in both inflammation and tissue repair: they triggered tissue repair (non-haemopoietic cells) and promoted differentiation into inflammatory cells such as neutrophils, eosinophils, basophils, mast cells, and monocytes. After tendon repair and reconstruction, moderate inflammation is critical for tendon/ligament healing, especially for macrophage polarization to help tissue remodeling, scar resolution, and immunosuppression [30]. In contrast, prolonged inflammation induces excessive remodeling and scar healing. In a comparison study, Lu et al. [31] injected autologous BM and BMSCs directly into the extra-articular tendon-bone tunnel in a rat model and found that increased M2 macrophage polarization in the autologous BM group resulted in more interfacial organization of fibers with increasing stiffness and failure load compared to the BMSCs group. In our study, BM cells caused higher MCP1 expression at two weeks post operation, indicating that both BMMNCs and BMSCs induced more inflammation factors in the early stage, which might reduce surrounding cell migration and proliferation in the graft with interfacial broader fibrocartilage formation after allograft ACLR. 

Growth factors and cytokines are important in tissue regeneration after ACL injury. Platelet-rich plasma (PRP) was shown to improve the quality of graft tissue, resulting in enhanced biomechanical properties and increased collagen expression in ACLR animal models [32,33]. Furthermore, PRP promotes tissue regeneration owing to its anti-inflammatory effects through different signaling pathways, release of multiple anti-inflammatory biomolecules, and modulation of macrophage subtypes [34,35]. Extracellular vesicles (EVs), paracrine factors from stem cells, have been applied as novel biomaterials in tissue regeneration. Shi et al. [36] demonstrated that EVs regulate BMSCs toward tenogenic differentiation, attenuate inflammation by increasing the expression of anti-inflammatory mediators, and enhance tendon healing in a patellar tendon injury model. Furthermore, EV delivery could increase M2 macrophage polarization and decrease M1 macrophage population as well as proinflammatory factors in the early phase of mouse Achilles tendon-bone reconstruction, improving tendon-bone healing [37]. The upregulated expression of MCP1 in our study indicated higher inflammation within the tendon-bone interface in the early stage after BM cell injection. Further studies are required to elucidate whether the combination of intra-articular BM cells and PRP or EVs could regulate early inflammation and enhance graft maturation and tendon-bone healing in allograft ACLR.

The present study has several limitations to it, including the lack of mechanical tests to investigate the ACL allograft-bone tunnel interfacial strength. In this study, we found progressive fibrocartilage maturation in the tendon-bone interface in both BMMNCs and BMSCs groups, in contrast to fibrous healing in the control group. Previous studies on the use of BM cells in the tendon-bone interface revealed progressive fibrocartilage maturation, leading to increased failure load in biomechanical tests [29,38]. In this study, we delivered only BM-derived cells through intra-articular injection. Different cell sources and delivery methods may help clarify the best approach to augment graft cell repopulation and interfacial healing after allograft ACLR. Small sample size and a short follow up period were some other limitations to our study. Therefore, further studies with larger sample sizes and longer follow up time are needed.

In conclusion, the regeneration capacity of freshly isolated BMMNCs is comparable to that of cultured BMSCs in rabbit allograft ACLR model. However, intra-articular BM cell injection had limited benefits on graft cell repopulation, led to higher inflammation in the early stage, with later progressive interfacial fibrocartilage maturation.

## 4. Materials and Methods

### 4.1. Animal Model

In this study, 14 skeletally mature New Zealand male rabbits weighing 2.5–3.0 kg were used. Allograft ACLR were performed in both knees of these rabbits. All rabbits with two operated knees were randomly divided into the experimental groups (fresh isolated BMMNCs or cultured BMSCs injection after ACLR; *n* = 5 knees per group, per time-point) and control group (without cell seeding after ACLR; *n* = 4 knees per time-point). Rabbits were euthanized at two different time-points: two and six weeks post surgery. All animal experiments were approved on 13 June 2017 by the Institutional Animal Care and Use Committee (IACUC 105263).

### 4.2. Harvesting Bone Marrow Stromal Cells 

Two weeks before the ACLR procedure, BMSCs were harvested from rabbit iliac crests. Bone marrow (8 mL) was aspirated from the iliac crest with a 20-mL syringe containing 2 mL heparin and gently mixed. The heparin and bone marrow solution was transferred into a 50-mL centrifuge tube and washed with 10 mL phosphate buffered saline (PBS). After centrifugation at 1500 rpm for 5 min at room temperature, 15 mL supernatant was removed and the cell pellet was resuspended in 25 mL cell growth medium containing minimal essential medium with Earle’s salts (GIBCO, Grand Island, NY, USA), 10% foetal calf serum (FCS), and 1% antibiotics (penicillin/streptomycin, GIBCO). Three equal aliquots of the resuspended bone marrow cells were seeded in 100-mm cell culture dishes and incubated at 37 °C in a humidified incubator with 5% CO_2_. BMSCs were grown as adherent cells and the culture medium was changed every three days. At 80–90% confluence, the cells were detached with 0.25% trypsin and sub-cultured. BMSCs at passage four were used for experiments in this study.

### 4.3. Preparation of Decellularized Tendon Graft

Hamstring tendons were harvested from rabbits euthanized in other IACUC-approved studies. The hamstring tendons were decellularized by freeze-thawing. Tendons were immersed in liquid nitrogen for 1 min and then thawed for 5 min in saline solution at 37 °C; this process was repeated five times. Next, the tendons were incubated in nuclease solution (RNase 100 μg/mL and DNase 150 IU/mL; Roche Diagnostic, Indianapolis, IN, USA) for 12 h at 37 °C, as described previously, to obtain an average DNA concentration of 11.5 ± 10.1 ng/mg decellularized tendon [39,40]. Finally, the tendons were rinsed three times with PBS for 30 min each at room temperature, with gentle agitation. The decellularized tendons were lyophilized for 24 h and gas sterilized using ethylene oxide. The lyophilized decellularized tendon grafts were stored at ambient room temperature. Prior to use in rabbit ACL surgery, the lyophilized decellularized tendon grafts were immersed in PBS for 24 h.

### 4.4. Harvesting Fresh Bone Marrow Cells

After anaesthetizing the rabbits, bone marrow (8 mL) was aspirated from the iliac with a 20-mL syringe containing 2 mL heparin. BMMNCs were isolated from the aspirated bone marrow by density gradient centrifugation. The bone marrow was diluted 1:1 in PBS and carefully overlaid on 15 mL Ficoll-Paque Plus (Amersham Biosciences, Uppsala, Sweden) in a 50-mL tube. The tubes were centrifuged at 1500 rpm for 30 min at 20 °C. After centrifugation, the top plasma layer was removed first and then the buffy coat was carefully transferred to a fresh 15-mL tube. The buffy coat layer containing BMMNCs was washed with PBS and centrifuged at 1000 rpm for 10 min at 20 °C; this was repeated twice. The suspension was discarded, the pellet was resuspended in culture medium, and cell number was counted.

### 4.5. PKH26 Labelled Cells

In order to monitor the seeded cells, freshly isolated BMMNCs and cultured BMSCs were labelled with PKH26 Red Fluorescent Cell Linker Kit (Sigma-Aldrich, St. Louis, MO, USA); 5 µL of the dye solution was added per mL of cell suspension (1 × 10^6^ cells/mL). After incubation at 37 °C for 20 min, the labelled cells were centrifuged at 1500 rpm for 5 min and re-suspended in culture medium. The labeled cells were prepared at a concentration of 1 × 10^7^ cells/100 µL PBS before injection.

### 4.6. ACL Allograft Reconstruction 

**Figure 7:**
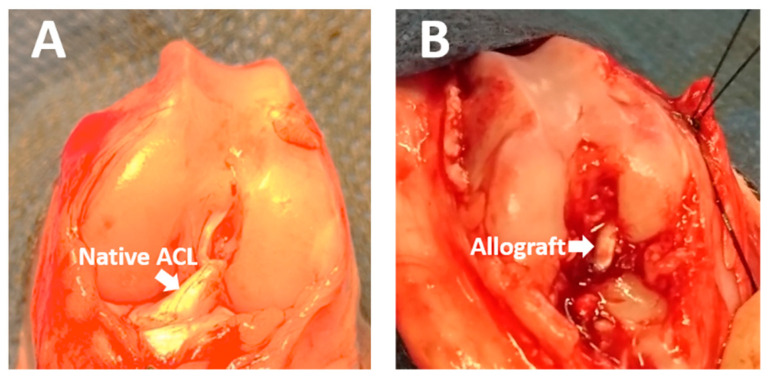
(**A**) The rabbit knee joint was exposed and the anterior cruciate ligament (ACL) was identified. (**B**) The allograft was passed through the tibial and femoral bone tunnel.

Rabbits were anesthetized with intravenously administered sodium pentobarbital (30 mg/kg). The knee joint was approached through a lateral parapatellar incision using aseptic techniques and the ACL was reconstructed with decellularized hamstring allograft. The allograft was trimmed to 2.0 mm diameter and the two free tendon ends were prepared with Bunnell suture using a 4-0 nylon suture. After retracting the anterior tibialis muscle laterally, a 2.0-mm bone tunnel was created at a 45° angle to the tibial axis and at 30° angle to the sagittal plane through the proximal tibial metaphysis to the footprint of the ACL stump with a 2.0-mm drill bit. The femoral tunnel was created through the femoral footprint with a 2.0-mm drill bit at 70° angle knee flexion. The prepared allograft was passed through the bone tunnel and fixed by suturing to the periosteum and surrounding soft tissue (Figure 7). The joint capsule, fascia, subcutaneous tissue, and skin were closed with 4-0 nylon suture. In the experimental groups, wither PKH26-labelled freshly isolated BMMNCs (1 × 10^7^ cells in 100 µL PBS) or cultured BMSCs (1 × 10^7^ cells in 100 µL PBS) were injected into the knee joints (*n* = 5 per time-point: two and six weeks after surgery in both BMMNC and BMSC groups). In the control group, the ACL was reconstructed with decellularized hamstring allograft without cell injection (*n* = 4 per time-point: two and six weeks post surgery). After surgery, rabbits were kept in individual cages without activity restriction. After two and six weeks, rabbits were euthanized via carbon dioxide inhalation. Knee joints were obtained and prepared for intra-articular graft and proximal tibia analyses. Intra-articular analysis investigated cellular repopulation in the allograft, while proximal tibia analysis evaluated tendon-bone tunnel interfacial healing.

### 4.7. Analysis of Cell Repopulation in the Intra-Articular Graft

#### 4.7.1. Histological Study

Intra-articular graft samples were prepared for histological analysis at two and six weeks post surgery. After fixation, the samples were embedded in paraffin and cut parallelly to the longitudinal axis of the graft. These samples were stained with haematoxylin and eosin (H&E) to examine collagen fiber and cell distribution in the graft.

#### 4.7.2. Total Cell Count

The slices were counterstained with DAPI (Vector Laboratories Inc, Burlingame, CA, USA) for nuclear staining and observed with confocal microscopy (Z2; Carl Zeiss, Oberkochen, Germany). Total cells (DAPI stained in blue) and seeded cells (PKH26 stained in red) in the allografts of the control, BMMNC, and BMSC groups were counted in three randomly selected fields (300 × 300 μm) of three samples per study group using ImageJ (64-bit Java v. 1.6.0_24; NIH, Bethesda, MD, USA) software and expressed as cell number per mm^2^.

### 4.8. Analysis of Tendon-Bone Tunnel Interfacial Healing in the Proximal Tibia

#### 4.8.1. Histological Study

Proximal tibia samples were prepared at two and six weeks post surgery. After fixation and decalcification, the samples were embedded in paraffin and cut cross-sectionally and parallelly to the tibial plateau surface for observing the tendon-bone tunnel interface. The samples were stained with H&E and Masson’s trichrome to examine the healing pattern within the interface of the tendon-bone tunnel. Samples from the BMMNC and BMSC groups (stained with PKH26) were collected at two and six weeks after injection with the respective bone marrow cells and observed under microscope (Leica CTR6000) to confirm cell infiltration in the tendon-bone interface.

#### 4.8.2. Micro–Computed Tomography (Micro-CT)

Micro-CT measurements (Skyscan 1272; Bruker. Billerica, MA, USA) quantified the mineralized tissue ingrowth around the bone tunnel and the status of tunnel widening. Each specimen (*n* = 4 in the control group, *n* = 5 in BM cells-injected groups at both two and six weeks) was scanned perpendicular to the long bone axis covering the entrance and exit of the tibial tunnel. We evaluated the cross section hole area, bone marrow density (BMD), percentage bone volume (Bone Volume (BV)/Total Volume (TV)), and bone surface density (BS/TV) with a CT Analyzer (version 1.16.1.0+ (64-bit)) software.

#### 4.8.3. Immunohistochemical Analysis 

To observe collagen formation and inflammatory cell infiltration, the proximal tibia samples were cut into 5-µm thick sections; IHC was performed with collagen I (1:200; Arigo Biolaboratories, Hsinchu, Taiwan) and monocyte chemoattractant protein-1 (MCP1) (1:200; Proteintech, Rosemont, IL, USA) antibodies. After antigen retrieval, the slides were washed twice with Tris-buffered saline (TBS) (Sigma-Aldrich, St. Louis, MO, USA) containing 0.025% Triton X-100 (TBST) for 5 min. The slides were blocked with IHC detecting kit (ab64264; Abcam, Cambridge, UK) for 1 h at room temperature according to manufacturer’s instructions. The primary antibodies were diluted in TBS with 1% BSA, incubated overnight at 4 °C, and then rinsed with TBST five times. The enzyme-conjugated secondary antibody was diluted in TBS with 1% BSA and incubated on the slides for 1 h at room temperature. The slides were counterstained with DAPI and observed under a light microscope (CTR6000; Leica Microsystems CMS GmbH, Wetzlar, Germany). The IHC results of collagen I and MCP1 were collected from three randomized areas per sample (*n* = 3 in each group at both two and six weeks) and analyzed with Image-Pro Plus 6.0 software (Media Cybernetics. Rockville, MD, USA).

### 4.9. Statistical Analysis

The results of cell number, Micro-CT, and IHC are shown as mean ± standard error of the mean (SE). Statistical analyses of the control, BMMNC, and BMSC groups were performed using one-way analysis of variance (ANOVA) with Tukey’s post hoc analysis and *p* < 0.05 was considered statistically significant.

## Data Availability

MDPI Research Data Policies.
